# Psychological Distress Factors among Caregivers of Children Receiving Cancer Therapy, at Muhimbili National Hospital

**DOI:** 10.24248/eahrj.v6i1.681

**Published:** 2022-07

**Authors:** Godfrey Malangwa, Ezekiel J Mangi

**Affiliations:** a Department of Sociology, College of Social Science, University of Dar es Salaam, Dar es Salaam, Tanzania; b Department of Behavioral Sciences, School of Public Health and Social Sciences, Muhimbili University of health and Allied Sciences, Dar es Salaam, Tanzania; c Muhimbili National Hospital-Mloganzila, Tanzania

## Abstract

**Background:**

Psychological distress is a major problem among caregivers of patients with chronic illness such as cancer. Psychological distress in caregivers affects the provision of healthcare services. The aim of this study was to determine the magnitude and factors contributing to psychological distress among caregivers of children receiving cancer treatment at Muhimbili National Hospital.

**Method:**

A cross sectional hospital-based study was done. Questionnaires were used to collect information on socio demographic characteristics and psychological support while Hopkins Symptoms Checklist 25 was used to determine psychological distress. The association between the socio demographic characteristics, psychological support and psychological distress was determined using logistic regression analysis (univariate and multivariate analysis) and p values of less than 0.05 were considered significant.

**Results:**

Prevalence of psychological distress was 64 (66.7%) among 96 caregivers interviewed while the proportion of psychological support was 71 (74%.) The social demographic characteristics were not associated with psychological distress. Psychological support from family member was protective against psychological distress (p=0.025, OR=0.047, CI 0.003-0.686) while other means of psychological support and self-copying mechanism to psychological distress were not associated with psychological distress.

**Conclusion:**

The prevalence of psychological distress was high despite the high proportion of psychological support. Socio demographic characteristics had no association with psychological distress. Psychological support from family member was protective against psychological distress. Further studies are needed to assess the social cultural aspect, content and context of psychological support.

## BACKGROUND

Psychological distress is a major problem among caregivers of patients with chronic illness such as diabetes, hypertension and cancer. The prevalence of psychological distress is 66% among caregivers of children with cancer while it was 65% in caregivers of spouse with cancer and 43% of caregivers of parent with cancer in the United Kingdom 2014.^1^ In 2016, the prevalence of parental distress was 56.0% of parents whose children were hospitalized for cancer treatment in Lebanon ^2^ and in Iraq 70.5% of parents whose children have cancer were depressed.^3^ In US 2013 the prevalence of psychological distress was over 50% among parents whose children had cancer and 16% had serious distress which needed immediate intervention while the national prevalence of psychological distress among population was 3%.^4^ In Uganda 45% of family members of patient with cancer had anxiety and 26% had depression in 2017.^5^

In Tanzania, approximately 100 new cases of cancer are diagnosed per 100,000 populations each year. The number of cancer patients attending specialized clinic for cancer (Ocean Road Cancer Institute) have almost doubled in 6 years from 2807 in 2006 to 5479 in 2013.^6^ In Tanzania the prevalence of common mental disorder including psychological distress is 48% among patients attending traditional healer clinics, 24% among patients attending primary health care facility^7^ and the characteristics of, those attending primary health care clinics (PHCs) and 6.5% among people with Asian origin living in Tanzania^8^ while in the general population it is 2.5%.^9^

Psychological distress is a result of unpleasant and harmful stress that becomes too great or lasts long that may result in negative feelings or reactions. Psychological distress occurs when body copying mechanism against bad stress begins to breakdown.^10^

Caregivers of patients with cancer are at high risk of psychological distress due to financial burden of cancer treatment because cancer treatment is very expensive.^11^ Psychological distress in caregivers will affect the provision of care to patients hence may affect management of the patient suffering from chronic illness such as cancer. Caring a child with chronic illness is more stressful situation than caring a healthy child.^12^ Also caring a child with cancer is highly stressful situation comparing to caring a spouse and a parent with cancer.^1^

The main reasons for the distress are perception of the parents about the prognosis of cancer, child's sufferings from symptoms like severe pain, weight loss and side effects of chemotherapy like excessive vomiting. The other reasons are general knowledge on the modality of treatment like radiotherapy, chemotherapy and surgery,^13^ high costs of treatment and most of cancer patients have poor prognosis especially when they are at late stage.^4^ The other factors for the psychological distress in caregivers of children with cancer are financial, employment status of the caregivers,^2^ sex of the care taker, number of hospital admissions and family support.^10^

It is important to consider psychological distress of the caregiver of the child because it is not only important for wellbeing of the caregivers but also may influence provision of service to the sick child. Thecaregivers play an important role in provision of physical and mental support to the child. Addressing the magnitude and contributing factors of psychological distress among caregivers of children with cancer in a scientific way will open eyes to the health practitioners and the community on the burden and factors associated with psychological distress among caregivers of children with cancer.

This study aimed to determine the magnitude and factors contributing to psychological distress among caregivers of children receiving cancer treatment at Muhimbili National Hospital in Dar es Salaam, Tanzania.

## MATERIAL AND METHODS

### Study Design

This was a hospital based cross sectionalstudy conducted for 30 days from 1^st^ July to 30^th^ July 2019.

Study Area The study was conducted at Muhimbili National Hospital (MNH) in Pediatric cancer ward and clinic. MNH is also a research center and university teaching hospital located in Dar es Salaam. It has 1,500 beds capacity, 1,000-1,200 outpatients per day and 1,000-1,200 patients are admitted per week. Pediatric cancer unit has an average of 90 inpatients and 38outpatientsper week, making a total of 128 patients per week. Patients with cancer stay longer in the ward than other patients because treatment of cancer takes along duration and needs in patient care. About 300-350 new cases are admitted each year in pediatric cancer ward.^14^

### Study Population

The study involved caregivers aging 18-60 years whose children were less than 18 years attending outpatient clinic or were inpatient at pediatric cancer unit at MNH and hadstarted treatment of cancer.

### Dependent Variable

Dependent variable was psychological distress.

### Independent Variable

Independent variables were socio demographic factors; age, sex, occupation, level of education, marital status, category of treatment, duration of cancer, longest time to stay in the hospital when admitted, number of hospital admission and living the same house. The other independent variables were type of psychological support, place of psychological support was given, number of times psychological support was given and self-copying mechanism.

### Inclusion Criteria

Caregivers with children confirmed with cancer.

### Exclusion Criteria

Caregivers who had experienced death of first degree relative or husband for the period of less than 6 months prior to the study and those who were on medication for mental disorders were excluded from the study because they could have psychological distress due to their situation which was not associated with the illness of their children.

### Sample Size Determination

Sample size of the quantitative cross-sectional study was calculated using the following sample size calculation formula.^15^ The prevalence of psychological distress was 4.8%.^16^ The sample size was 73 caregivers. Adjusting to non-response rate which is 4% in similar study in Uganda.^5^ The minimum sample size was 96 caregivers.

### Data Collection Methods and Instruments

Caregivers were randomly selected from the clinic and in the ward. Data were collected by interview method using structured questionnaire and Hopkins Symptoms Checklist (HSCL-25) tool. Structured questionnaire was used first to collect information on demographic characteristic, social cultural factors and psychological support then HSCL-25 tool was used to determine psychological distress.

HSCL-25 has 25 items, 10 for anxiety and 15 for depression. Each item has a score from 1 to 4 indicating that whether the symptom was not at all, a little, quite a bit or extreme present, respectively. HSCL-25 score was calculated by dividing the sum of the score of individual items by the number of answered items. The score obtained can be used for determining of psychological distress. The respondent was considered has psychological distress if the middle value of HSCL-25 was ≥1.55 and the value of ≥1.75 was severe psychological distress which requires treatment.

The study done previously in Tanzania, shows that HSCL-25 has not only good consistency, good inter-rater reliability and test retest reliability but also valid on content, construct and discriminant methods.^18^ It has a sensitivity 89% and specificity of 80% when compared toDiagnostic and Statistical Manual of Mental Disorders (DSM) IV.^19^ a two-phased design included measures for health-related quality of life, perceived social support, and the HSCL-25 screen for depressive (HSCL-15 subscale It also has a sensitivity of 88% and specificity of 73% for measuring DSM III major depression.^20^

Pretest of the questionnaire was done before data collection. Data were collected by two trained research assistants using translated Kiswahili version of the questionnaire.

### Data Analysis

Data were coded and entered in Statistical Product and Services Solution (SPSS) version 22 which is the property of International Business Machine (IBM) cooperation licensed 2011. Descriptive Statistics (Descriptive Analysis) was used to find the proportion of psychological distress.

Categorical variables were summarized by using proportions and quantitative variables were summarized by using measures of central tendency.

The mean, percentage, proportion and frequency were also used to summarize data from the questionnaire and HSCL-25. Data were cleaned before analysis so as to identify the incorrect and missing data. Logistic regression (univariate and multivariate analysis) were done to determine the association of independent variable and dependent variables. The *P* value of less than 0.05 was considered significant.

### Ethical Consideration

Ethical approval was obtained from Muhimbili National Hospital with certificate number MNH/IRB/II/2019/018

The participation was on voluntary basis; caregivers were not given anything as a token for participating in the study. Caregivers signed consent form before participating in the study.

In case of unexpected outcomes like emotional or psychological difficulties, participants were directed to the nurse who was on duty at the clinic or in the ward for support. Caregivers were allowed to withdraw from or not to participate in thestudy and they were assured that withdrawal will not affect treatment of the patient. Confidentiality was assured to all participants.

## RESULTS

### Demographic characteristics

A total of 96 caregivers were interviewed of whom 70 (72.9%) were female. The age of the caregivers varied from 18-60 years. The majority of the cancer caregivers were peasants 39 (40.6%) or self-employed 41 (42.7%) while a small percentage were employed by the government 1 (1%) or private sector 2 (2.1%). Other demographic characteristics are shown in Table 1.

**TABLE 1:** Demographic Characteristics of the Study Population

Characteristics	Frequency (n=96)	Mean
**Sex of the caretaker**		
Male	26 (27.1%)	
Female	70 (72.9%)	
**Age group of the caretaker (in years)**		
18–24	11 (11.5%)	
25–34	34 (35.4%)	35.9 years
35–44	31 (32.3%)	
45–60	20 (20.8%)	
**Sex of the child**		
Male	53 (55.2%)	
Female	43 (44.8%)	
**Marital status of the caretaker**		
Single	9 (9.4%)	
Married	73 (76.0%)	
Cohabiting	4 (4.2%)	
Divorced	5 (5.2%)	
Widow	5 (5.2%)	
**Age group of the child (in years)**		
< 4	36 (37.5%)	
5–11	43 (44.8%)	
12–14	14 (14.6%)	
15–17	3 (3.1%)	
**Relationship with the child**		
Father/Mather	79 (82.3%)	
Brother/Sister	8 (8.3%)	
Aunt/Uncle	3 (3.1%)	
Grandmother/father	6 (6.3%)	
**Living the same house status**		
Yes	89 (92.7%)	
No	7 (7.3%)	
**Level of education**		
Not attended school	11 (11.5%)	
Primary education	62 (64.6%)	
Secondary education	20 (20.8%)	
University education	3 (3.1%)	
**Occupation**		
Housewife	13 (13.5%)	
Peasant	39 (40.6%)	
Employed in-	2 (2.1%)	
private sector		
Employed in-	1 (1.0%)	
government sector		
Self employed	41 (42.7%)	

### Prevalence of Psychological Distress

The prevalence of psychological distress among caregivers of children receiving cancer treatment was 64(66.7%) of the study population using HSCL-25.

### Variation of psychological distress by social demographic characteristics.

The proportion of psychological distress was 48(68.6%) and 16(61.5%)among female and male caregivers, respectively. However, the difference of psychological distress according to sex of caregiver was not statically significant (OR0.733; 95% CI 0.287 to 1.873; *P*=.517). The proportion of psychological distress among inpatient caregivers was 42(65.6%) while it was 68.8% (22 out of 32) among outpatient caregivers. However, the difference of psychological distress according to category of treatment; either inpatient or outpatient (OR0.868; 95% CI 0.350 to 2.152; *P*=.770) was not significant. Other results are shown in Table 2.

**TABLE 2:** Association Between Social Demographic Characteristics and Psychological Distress

Factors	Univariate Analysis			Multivariate Analysis		
	cOR	CI (95%)	P Value	aOR	CI (95%)	P Value
**Sex of caregiver**						
Male	0.733	0.287–1.873	0.517			
Female	Ref					
**Category of treatment**						
Inpatient	0.868	0.350–2.152	0.770			
Outpatient	Ref					
**Age**						
18–24	Ref					
25–34	2.000	0.494–8.069	0.331			
35–44	2.037	0.493–8.408	0.325			
45–60	1.250	0.283–5.525	0.769			
**Marital**						
Single	Ref					
Married	1.856	0.454–7.571	0.389			
Cohabiting	0.800	0.076–8.474	0.853			
Divorce	3,200	0.248–41.208	0.372			
Widow	0.533	0.580–4.912	0.572			
**Relationship with the child**						
Father/Mather	Ref					
Brother/Sister	0.463	0.107–2.003	0.303			
Aunt/Uncle	0.231	0.020–2.674	0.241			
Grandmother/father	2.316	0.257–20.865	0.454			
**Living the same**						
YES	Ref					
NO	0.644	0.135–3.070	0.581			
**Level of education**						
Not attended school	Ref					
Primary education	1.750	0.477–6.438	0.399			
Secondary education	1.548	0.345–6.942	0.568			
University education	13462	29054 0.000	0.999			
**Occupation**						
House wife/staying at home	Ref					
Peasant	0.711	0.186–2.724	0.619			
Employed in-	0.444	0.022–9.032	0.598			
private sector						
Employed in government	7179	0.000	1.000			
	8882					
	8.5					
Self employed	1.074	0.277–4.170	0.918			
**Duration of diagnosis of cancer (in months)**						
1–3	Ref		Ref			
4–6	0.452	0.143–1.431	0.177	0.477	0.123–1.851	0.285
7–12	0.928	0.270–3.192	0.905	1.107	0.264–4.646	0.890
13–24	0.464	0.123–1.753	0.257	0.421	0.089–1.986	0.274
Above 24	0.696	0.106–4.552	0.705	0.962	0.100–9.219	0.973
**Number of admissions**						
Never admitted	Ref					
1–2	2.043	0.270–15.442	0.489			
3–5	1.833	0.204–16.612	0.589			
Above 5	4.000	0.211–75.659	0.355			
**Longest duration of staying in the ward when admitted (days)**						
4–7	Ref		Ref			
8–14	1.667	0.074–37.728	0.748	2.036	0.086–48.208	0.660
15–30	2.778	0.367–21.029	0.323	8.417	0.323–219.212	0.200
30–60	10.00	1.260–79.339	0.029	2.540	0.125–51.427	0.544
61–150	3.667	0.619–21.730	0.152	2.425	0.117–50.055	0.566
151–210	2.738	0.565–13.267	0.211	6.201	0.212–181.428	0.289
Above 210	6.667	0.809–54.957	0.078	0.774	0.028–22.974	0.889

Abbreviations: aOR, adjusted odds ratio; cOR, cumulative odds ratio; CI, confidence interval

### Variation of Psychological Distress by Psychological Support

Caregivers were asked, if they hadreceived psychological support since their children were diagnosed with cancer. In this study, 71 (74%) of caregivers received psychological support since the time of diagnosis of their children. Among those who received psychological support 46(64.8%) had psychological distress (OR=1.40; 95% CI=0.51 to 3.80; *P*=.51) but the results were not statistically significant.

The proportion of psychological distress were higher 47(64.4%) among those patients who received counselingand those caregivers who allowed to meet with other caregivers as a way to prevent or reduce psychological distress 3(60%) but the difference was not significant (OR=1.22; 95% CI=0.191 to 7.83; *P*=.83).

Psychological distress varies according to the person who provides psychological support. Caregivers who received psychological support from family member were protected against psychological distress, only 2 (38.6%) had psychological distress and the results were significant (OR=0.047;95% CI=0.003 to 0.686; *P*=.025). However, the proportion of psychological distress among those who received psychological support from friend/neighbor was 7(70.0%)with OR=1.342; 95% CI=0.316 to 5.700; *P*=.69, and psychologist 3(75%) with OR=1.705; 95% CI=0.168 to 17.27; *P*=.65 were not significant. Other results are shown in Table 3.

**TABLE 3:** Association Between Psychological Support and Psychological Distress

Factors	Univariate Analysis			P Value	aOR	Multivariate Analysis	
		cOR	CI (95%)			CI (95%)	P Value
**Received psychological support**							
YES		1.40	0.51–3.80	0.51			
NO		Ref					
**Type of psychological support**							
Meeting with other caregivers	YES	1.22	1.19–7.83	0.83			
	NO	Ref					
Counseling	YES	2.00		0.001			
	NO	Ref					
**Place of psychological support**							
Cancer Unit		Ref					
Cancer unit and other hospital		0.147	0.014–1.509	0.107	0.000	0.000	0.998
Cancer unit and home		0.442	0.058–3.374	0.431	0.302	0.038–2.40	0.258
All of them		0.221	0.019–2.587	0.229	3.780	0.127–112.467	0.442
**Person who provides psychological support**							
Family member	YES	0.187	0.033–1.042	0.056	0.047	0.003–0.686	0.025
	NO	Ref					
Friend/Neighbor	YES	1.342	0.316–5.700	0.690			
	NO	Ref					
Doctor/Nurse	YES	0.000	0.000 1.000				
	NO	Ref					
Psychologist	YES	1.705	0.168–17.27	0.652			
		NO	Ref				
**Time taken to receive first psychological support in days**							
1–3 days		Ref					
4–7 days		0.244	0.041–1.450	0.121	0.185	0.029–1.199	0.077
8–14 days		7870	0.000	1.000	4879	0.000	1.000
		2621		1797			
		6.7		8.8			
15–30 days		7870	0.000	0.999	3.045	0.000	0.998
		2621		E+26			
		6.7					
31–60 days		0.244	0.021–2.858	0.261	0.092	0.005–1.777	0.114
Above 60 days		0.974	0.083–11.43	0.984	1222	0.000	0.999
					6453		
					9.8		
**Number of times psychological support**							
1		Ref					
2–3		0.824	0.210–3.234	0.782			
4–7		0.714	0.179–2.843	0.633			
More than 7		0.330	0.073–1.479	0.147			
**Self-coping strategy with distress**							
Stay alone	YES	1.079	0.422–2.759	0.84			
	NO						
Sleeping	YES	1.376	0.444–4.270	0.580			
	NO						
Recreation	YES	0.644	0.135–3.070	0.581			
	NO						
Drinking alcohol	YES	8205	0.000	1.000			
		5866					
		2.0					
	NO						
Watching TV/Sport	YES	1.525	0.152–15.26	0.720			
	NO						
Talking to friends	YES	1.162	0.456–2.957	0.753			
	NO						
Praying	YES	1.398	0.514–3.798	0.512			
	NO						
Drinking water	YES	2.067	0.221–19.29	0.524			
	NO						

Abbreviations: aOR, adjusted odds ratio; cOR, cumulative odds ratio; CI, confidence interval

## DISCUSION

### Magnitude of psychological distress

The findings on the prevalence of psychological distress using HSCL-25 are consistent with those reported in the study done in the United Kingdom where the prevalence was 179(66.7%).^1^ However, the prevalence is higher compared to that from other studies; 54(45%) in Uganda,^5^ 64(56.0%) in Lebanon^21^ and 43(50%) in US^4^ but is lower as compared with the study done in Iraq which showed prevalence of 236(70.5%).^3^ The difference might be due to the fact that this study was conducted in a hospital setting which increase the risk of having psychological distress as compared to other studies which were conducted in the community.

### Social demographic characteristics

Psychological distress according to sex was not statistically significant. This result is in agreement with findings reported in a cross sectional study on distress in cancer patients and their caregivers, which showed that sex of caregivers had no association with psychological distress.^22^ 189 pairs of cancer patients (31% gastrointestinal, 34% lung, 35% urological cancers However different studies show the association of psychological distress according to sex., female caregivers were positive associated with psychological distress.^1,11,23,24^ psychological distress, fatigue, and quality of life (QOL The difference of the finding on sex, might be due difference in culture which varies on howto handle psychological distress.

There is no association of age with psychological distress. This result is similar to that from a study on psychological distress of female caregivers and significant others.^1^ Other social demographic characteristics such as marital status, relationship with the child, education level, occupation, duration of caregiving had no association with psychological distress and resemble those from a descriptive hospital based study done on caregivers of patients with schizophrenia.^25^

There is no association with other social factors such as living in the same house, number of hospital admissionsand duration of stay in the hospital and psychological distress of caregiver. However, in other studies, social demographic characteristics were associated with psychological distress; marital status,^23^ duration of illness,^2,11,26,27^ employment status,^11,24,28,29^ number of hospital admissions,^24^ relationship and bondage with the patient,^11^ and education level.^11^ The results of social demographic characteristics of having no association with psychological distress among caregivers of children receiving cancer treatment at MNH may be due the fact that treatment, meals and accommodation are given for free for the whole duration oftreatment which softens the social economic burden hence making caregivers less susceptible to psychological distress.

### Psychological Support

The study showed that, 71(74%) of caregivers received psychological supportand 46(64.8%) of caregivers with psychological distress received psychological support while psychological distress among caregivers with advanced cancer patients the proportion of caregivers who received psychological support was50(25%)^30^

The higher proportion of caregivers who received psychological support might be due to the fact that psychological support of the caregivers has been integrated in the management of children with cancer at MNH.

Psychological support from family member was protective against psychological distress. This result is in accordance with findings from many studies.^1,24,31^ This might be due to the reason that family members know each other well in terms of strength and weakness, time used when providing psychological support and they are all concerned with the situation hence this leads to successful provisionof psychological support.

In this study, self-coping mechanisms (stay alone, sleeping, recreation, drinking alcohol, watching TV/sports, talking with friend, praying and drinking water) were not associated with psychological distress. However, in other studies, there were associations of psychological distress and spiritual supportbeing protective,^32^ avoidance method being a risk,^11,33^ self-directed coping strategies being risk^24^ and problem solving being protective.^34^ This difference of this study and other studies might be due of different culture which may affect self-copying mechanism.

### Study Limitation

The study was conducted in hospital setting making caregivers being at increased risk of psychological distress as compared to community setting. The study used only one validated screening tool (HSCL-25) to determine psychological distress without assessing the clinical presentation which may cause over rating or under rating the psychological distress.

## CONCLUSION AND RECOMMENDATIONS

Despite claims that socio demographic characteristics and self-copying mechanism contribute to psychological distress of the caregiver; our study revealed that the only contribution to psychological distress of the caregiver was psychological support provided by the family member which was protective. Also, the study revealed that there is both high prevalence of psychological distress and proportion of caregivers who received psychological support.

Due to high prevalence of psychological distress and high proportion of caregivers who received psychological support, further studies are needed to assess the content and context of psychological support. Further studies are also needed to assess cultural influence of psychological distress of the caregiver such as self-copying mechanism which varies across different ethnicity.

Psychological support provided by family member was protective against psychological distress while psychological support from doctor/nurse or psychologist had no association with psychological distress; studies are needed to assess the time spent by health professional on providing psychological support.

This study used quantitative methods of data collection through validated tool in assessing psychological distress; further qualitative studies are needed to assess social cultural content and context of caregiver.
